# Case Report: Interparietal hernia due to posterior rectus sheath dehiscence following a Rives–Stoppa repair for a ventral hernia

**DOI:** 10.3389/fsurg.2026.1715780

**Published:** 2026-02-09

**Authors:** Moysis Moysidis, Ioannis Pliakos, Angeliki Chorti, Stavros Panidis, Theodossis Papavramidis

**Affiliations:** 1 Department of Minimally Invasive Surgery, Kyanous Stavros, Thessaloniki, Greece; 2 Propaedeutic Department of Surgery, AHEPA University Hospital, Aristotle University of Thessaloniki, Thessaloniki, Greece

**Keywords:** hernia, diastasis recti, Rives–Stoppa, ventral abdominal hernia, abdominal wall

## Abstract

**Introduction:**

Primary ventral abdominal wall hernias, especially when complicated by concomitant rectus abdominis diastasis, pose a complex challenge for surgeons. The retro-muscular Rives–Stoppa technique is a well-established and effective repair method. However, a rare but severe complication is the development of an interparietal hernia.

**Case presentation:**

We present the case of a 57-year-old male with an epigastric hernia and diastasis recti who underwent an open retro-muscular Rives–Stoppa repair. Postoperatively, he developed a series of non-specific symptoms including mild discomfort, nausea, and vomiting, without clear signs of hernia recurrence. On postoperative day four, his condition worsened with projectile bilious vomiting and acute kidney injury. A CT scan revealed an incarcerated interparietal hernia containing small bowel between the posterior rectus sheath and the mesh. He underwent an emergency reoperation to reduce the bowel and repair the defect. The patient had an uneventful recovery and was discharged on postoperative day seven.

**Discussion:**

Interparietal hernias are a rare complication of the Rives–Stoppa repair, resulting from dehiscence of the posterior rectus sheath. This can lead to incarcerated bowel without the external signs of a recurrent hernia, delaying diagnosis. High clinical suspicion and a low threshold for CT imaging are crucial. We review the current literature, highlighting the scarcity of reported cases and the various surgical approaches, which include open, laparoscopic, or expectant management.

**Conclusion:**

Although uncommon, interparietal hernia should be considered in any patient who fails to thrive after a Rives–Stoppa repair. This case emphasizes the need for prompt diagnosis and a tailored management strategy to prevent severe morbidity.

## Introduction

Primary ventral abdominal wall hernia is a health condition that if left untreated may develop in acute complications and even mortality in the event of bowel incarceration. Although this may not always be the case, its natural development is that of worsening discomfort, pain, reduced quality of life (1). When combined with diastasis recti, it constitutes a complex condition for both the patient and the physician. For the patient, cosmetic as well as quality of life reasons will be alarming and make them seek medical assistance. On the other hand, for the surgeon, even a small actual ventral defect may need a complex abdominal wall reconstruction when diastasis recti co-exist.

As of today, there is no unanimity regarding the proposed repair of a concomitant rectus diastasis with an epigastric hernia, but all evidence points towards a mesh repair with abdominal wall reconstruction (2) As for a medium sized, uncomplicated, epigastric hernia (M1-2, W2, on the European Hernia Society Classification), one of the most popular technics is the retro-muscular approach, as described by Rives and Stoppa (3, 4). Outcomes with the use of this procedure are very favorable and overall short-term severe morbidity and mortality rates are very low (2.7% and 0.2% respectively). Early reports from the minimally invasive Rives–Stoppa technic are even more promising. Moreover, its safety profile is superior to both on-lay and intraperitoneal mesh placement (5, 6).

Interparietal hernia of the retro-muscular plain is a rare complication of the procedure and can occur after both open and minimally invasive approach (7). Although not common, it can be a severe complication accompanied with bowel obstruction, without clinical signs of herniation. The scope of this case presentation is to focus attention on the matter, raise clinical suspicion and discuss all possible intervention choices.

## Case presentation

A 57-year-old male with a history of hypertension, Asperger's syndrome, and heavy smoking presented with an M2W1 epigastric hernia and rectus abdominis diastasis. Due to his occupation involving heavy lifting, he experienced daily pain and discomfort. A computed tomography (CT) scan confirmed the clinical findings, and he was scheduled for surgery.

He underwent an open retro-muscular hernia repair using the Rives–Stoppa technique with a 18 by 25 cm monofilament polypropylene mesh. The mesh was fixated using two interrupted stitches prolene 2-0, cranially and caudally. Both posterior and anterior rectus sheath were approximated using extra long-term absorbable monofilament synthetic suture made of poly-4-hydroxybutyrate (Monomax® 2-0). During the approximation care was taken to abide by the principles of the small bites' technique. Distance between each bite did not exceed 5–6 mm and each stitch involved tissue 5–8 mm wide. It was calculated that the suture length to wound length ratio was approximately 4:1. The repair was concluded without extensive tension and there was no need for a repeat dose of neuromuscular blocking agents (NMBAs) to complete the operation. He was discharged on the first postoperative day, fully mobile, and tolerating a solid diet. At discharge, he was afebrile, pain-free, and asymptomatic.

On postoperative day two, he reported mild discomfort, nausea, and loss of appetite. These symptoms worsened on day three, and by postoperative day four, he developed projectile bilious vomiting and abdominal distension. He insisted he had no pain, and his surgical wound appeared unremarkable. He remained mobile, refraining from any strenuous activities. Nevertheless, he complained of mild cough, which for him, as a smoker, was not out of the ordinary. Clinical examination revealed no signs of hernia recurrence. Laboratory tests showed acute kidney injury with a creatinine level of 5.48 mg/dL and urea of 116 mg/dL. His white blood cell count was also elevated at 17,000 cells/microliter.

**Figure 1:**
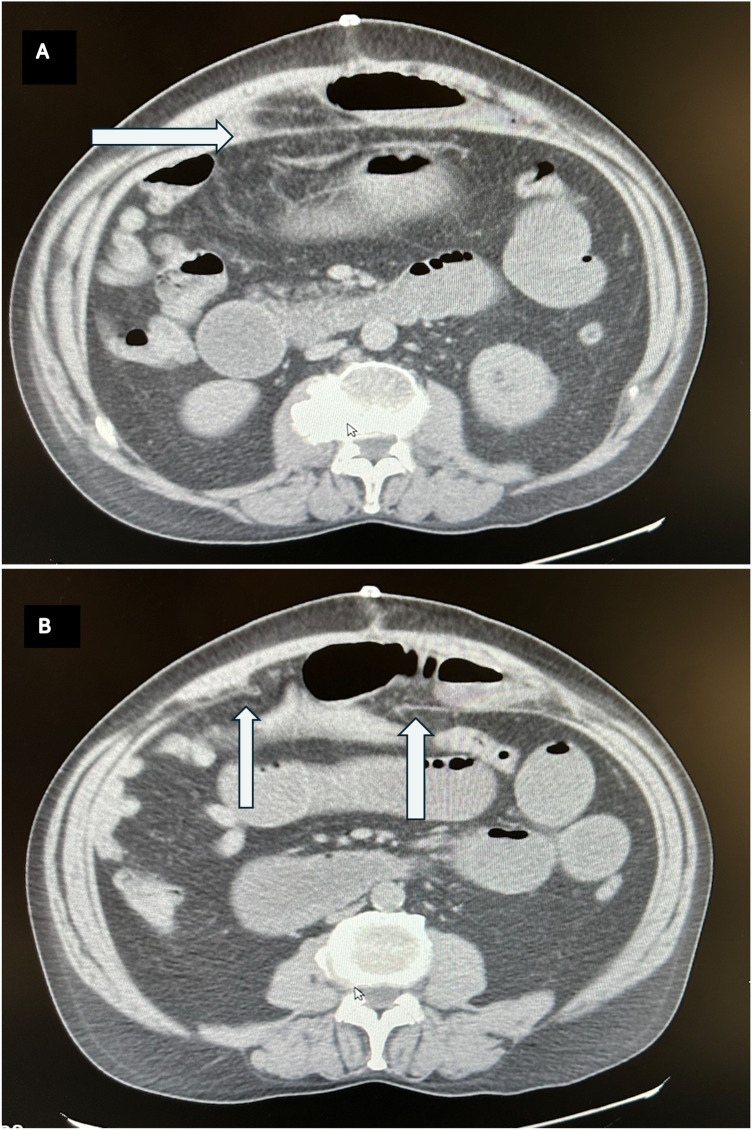
**(A,B)** Abdominal CT scan images showing the interparietal hernia. White arrows: The posterior rectus sheath. **(A)** Intact, **(B)** free edges surrounding the orifice.

A repeat CT scan was performed, which revealed an interparietal hernia with small bowel content incarcerated between the posterior rectus sheath and the mesh. The mesh was correctly positioned with no signs of migration, and there was no disruption of the anterior rectus sheath. The herniated bowel was dilated with a clear transition point. No free air or fluid was present (Figure 1).

A nasogastric tube was inserted to decompress the stomach and relieve the vomiting. He was started on intravenous fluid resuscitation and monitored for hourly urine output, as he was anuric for the first three hours of his readmission.

**Figure 2:**
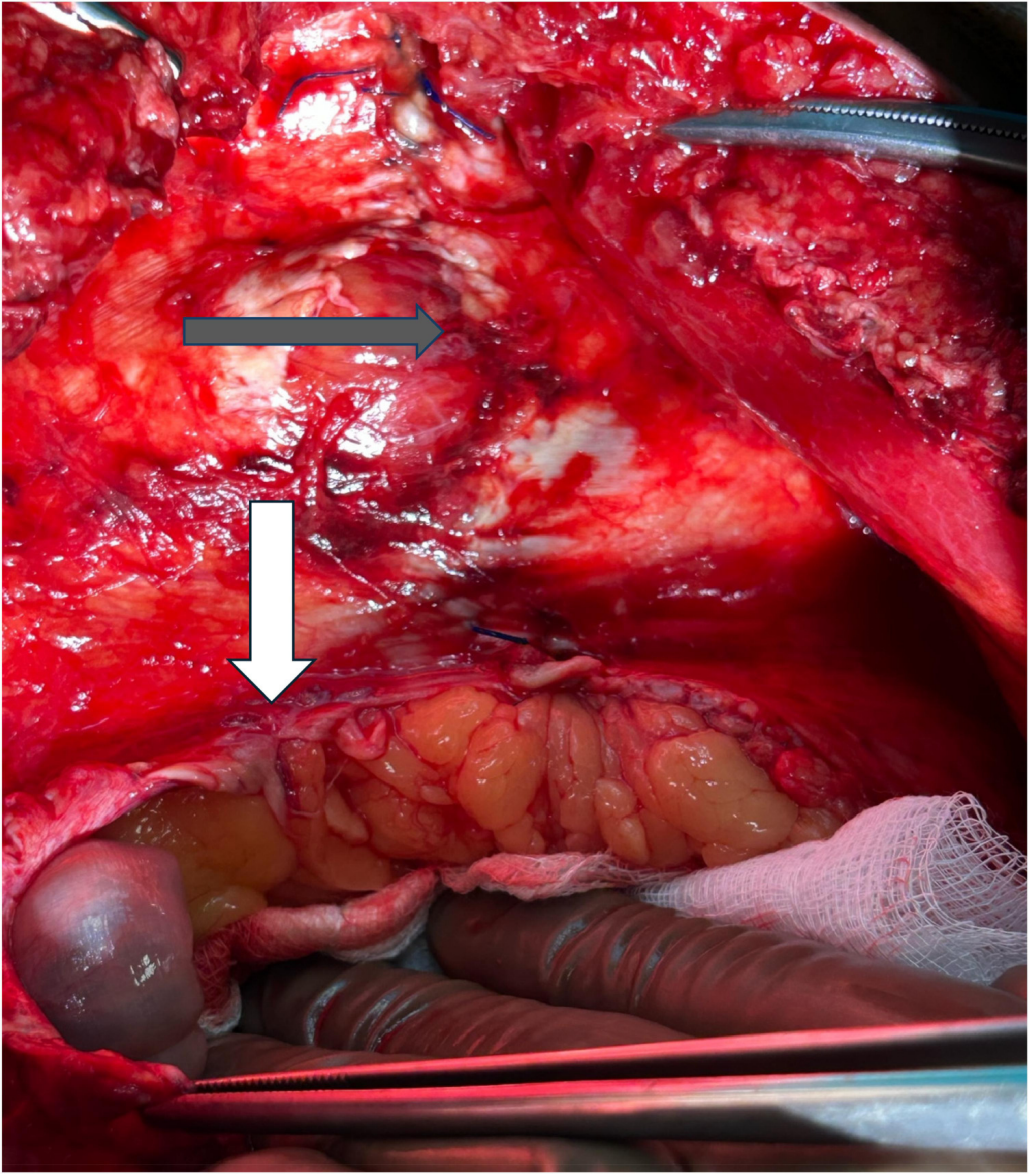
Intraoperative picture showing the defect of the posterior rectus sheath. Grey arrow: intact closure of the posterior rectus sheath. White arrow: Hernia orifice.

He subsequently underwent an emergency open reoperation. The surgery confirmed the entrapment of bowel loops between the posterior rectus sheath and the mesh. The bowel loops were distended, and the transition point was found after removing the mesh. No folding or mesh migration was detected and there were no adhesions or signs of integration between the mesh and the surrounding tissues. A 5 cm opening was found at the lower end of the posterior rectus sheath (Figure 2). The defect was primarily repaired, and the remaining portion was sutured to the rectus muscle. A new mesh was not placed due to the risk of contamination.

The patient had an uneventful postoperative recovery. His kidney function returned to normal, and his bowels opened on postoperative day three. He was discharged the following day, free of symptoms. At his follow-up appointment on postoperative day 12, his surgical clips were removed, and the wound was unremarkable. He was advised to avoid heavy lifting for three months. No signs of recurrence were detected on the three-month follow-up.

## Discussion

Complications after a retro-muscular repair are rare, most notably surgical site infection, seroma formation and early or late recurrence. Mortality rates post a Rives–Stoppa procedure is extremely rare (1, 5). The quality of life of patients undergoing such procedures is considerably improved (8). Moreover, those results are comparable between both open and minimally invasive approach (9). One of the least discussed about, albeit grave complication, is interparietal hernia on the retro-muscular plain. In a recent retrospective cohort study of laparoscopic enhanced view totally extra-peritoneal Rives–Stoppa (eTEP-RS) repair of incisional ventral hernias in obese patients, it was found that 9.6% (13 out of 135) of the patients developed posterior rectus sheath dehiscence (10). This rare and possibly lethal complication may cause bowel incarceration and small bowel perforation without clinical signs of a recurrent hernia, as both the mesh and the anterior sheath is intact. This paradox may lead to delayed diagnosis with possible adverse outcomes.

Interparietal hernia comes as a result of the dehiscence of the posterior rectus sheath. This is mainly due to excessive tension, and may be avoided by a number of techniques, such as transversus abdominis release or preoperative botulinum toxin injection for large ventral hernias with loss of domain. Factors contributing to this complication may be severing coughing, smoking, obesity, post-operative ileus, vomiting, and intensive exercise. In a handful of cases in the literature, postoperative interparietal hematoma or seroma may have been the cause of posterior sheath dehiscence and interparietal hernia (11, 12). In the case in discussion, a few factors that may have contributed to this condition have been identified. The patient suffered from chronic cough due to smoking, which he ceased only a few days before the operation. The combination of increased BMI and reduced mechanical strength of the abdominal wall tissues caused by diastasis recti has no doubt also weighed in. His immediate post op recovery from anaesthesia was complicated by a short episode of disorientation and, lastly, noncompliance with post op instructions as reported by his environment, are all probable causes of this adverse event.

High index of suspicion is required for the diagnosis of interparietal hernia because both the mesh and the anterior sheath remain in place, therefore there are no clinical signs of herniation. The main symptoms are abdominal distention, ileus, retching or vomiting. Pain may or may not be present. In some cases when bowel obstruction symptoms are not present, the diagnosis may be delayed (11, 13). In any case, one needs to have a low threshold for abdominal CT scan when a patient fails to thrive post a Rives–Stoppa repair. CT scan findings may be typical of bowel obstruction with dilated small or large bowel loops and a transition point, alongside herniation in the space between the posterior rectus sheath and the rectus muscle. Experienced clinicians may be able to distinguish the posterior sheath extending from its lateral attachments (12–14). Ultrasound as a diagnostic modality is not widely used as it is unlikely to identify interparietal herniation and may delay diagnosis with false negative results.

As soon as the diagnosis is reached, emergency reoperation is warranted. It is a fact that reported cases of interparietal hernias post a Rives–Stoppa repair are scarce in literature. In addition, most of them have wide differentiations, and as a result there is no current consensus on the proposed approach. This is the literature gap that this report would like to fill, with the presentation of all the techniques used so far and the rational behind each. In the review of the available literature, only 27 cases were reported (7, 10–13), including this. Of them, 13 refer to a study on ventral hernias on obese patients and there is no mention of the type of repair (10). The remaining 14 are presented in more detail (Table 1).

**Table 1:** All reported cases of interparietal hernias due to posterior rectus sheath dehiscence.

Study	Open	Laparoscopic	Untreated	Not mentioned
Carbonell (11)	1	1		
Davis et al. (12)	1	6	2	
Honma et al. (13)		1		
Tanioka et al. (7)	1			
Rayman et al. (10)				13
Moysidis et al., 2026	1			
**Overall *N*** **=** **27**	**4 (14.8%)**	**8 (29.6%)**	**2 (7.4%)**	**13 (48.1%)**

It is worth mentioning that two of the reported cases by Davis et al. were found incidentally by CT scan performed for other reasons and were left untreated, as they were asymptomatic (12). This finding is an indication that the actual incidence of posterior rectus sheath dehiscence may be more common but doesn't present always with symptoms. Another approach used to repair this Interparietal hernia in literature is by open surgery. It may not be the most common, but it is the proffered method if a perforation has occurred, is suspected or pending, thus exposing the mesh to bowel content. Mesh migration is another reported reason to opt for the open approach. In the case of open repair, the preexisting mesh is most often removed, and the abdomen is closed in layers with or without the placement of a new mesh (7, 11, 12).

Finally, the most common technique used for interparietal hernias is the minimally invasive one. The main reasoning behind the technic is to reduce the herniating bowel and obliterate the defect without interfering with the overlying mesh, muscle and anterior rectus sheath. In the reviewed cases, three types of laparoscopic repairs are proposed. The first includes closing the orifice by approximation of the posterior rectus sheath with single transmural sutures when possible (13). The next approach employs the placement of an absorbable polyglactin mesh in an intraperitoneal position to cover the defect. Last but most common proposed technique is the fixation of the edges of the posterior rectus sheath to the overlying mesh and muscle with the use of tacks or sutures. This technique eliminates the potential space for recurrence but in the same time leaves part of the mesh exposed to the viscera (7, 11, 12). No data was provided regarding long term outcomes of each method. Consequently, careful consideration is needed in order to select the appropriate approach for each patient.

## Conclusion

Interparietal hernias following a Rives–Stoppa repair, while rare, are a critical complication that can present with non-specific symptoms and a high risk of bowel incarceration. A high index of suspicion is paramount for a timely diagnosis, which is often confirmed by CT imaging. The treatment approach should be individualized, with emergency reoperation as the mainstay of management. This case highlights the importance of recognizing this uncommon but serious complication and contributes to the limited literature on its diagnosis and surgical management.

## Data Availability

The raw data supporting the conclusions of this article will be made available by the authors, without undue reservation.
